# Structural and mechanistic insights into the catalytic versatility of lysyl hydroxylases in collagen post-translational modifications

**DOI:** 10.1016/j.jbc.2026.113259

**Published:** 2026-06-18

**Authors:** Susovon Ghosh, Swapnila Pramanick, Dilip Kumar, Bhaskar Mondal, Trayambak Basak

**Affiliations:** 1 School of Chemical Sciences, Indian Institute of Technology (IIT) Mandi, Mandi, Himachal Pradesh, India; 2 School of Biosciences and Bioengineering, Indian Institute of Technology (IIT) Mandi, Mandi, Himachal Pradesh, India; 3 Trivedi School of Biosciences, Ashoka University, Sonipat, Haryana, India; 4 Center for Human-Computer Interaction (CHCi), Indian Institute of Technology (IIT) Mandi, Mandi, Himachal Pradesh, India

**Keywords:** lysyl hydroxylase, collagen, catalysis, post-translational modifications, lysyl-hydroxylation, lysyl-O-glycosylation

## Abstract

Iron-dependent lysyl hydroxylases (LHs) play a pivotal role in collagen biosynthesis by catalyzing post-translational modifications (PTMs) of lysine residues. These PTMs involve site-specific hydroxylation and sequential O-glycosylation, facilitated either by LHs or by collagen galactosyltransferase (COLGALTs) enzyme complexes. Here, we present the indispensable evolutionary significance of the LH-catalyzed PTMs, *i.e.*, collagenous lysine hydroxylation (HyK) and lysyl-O-glycosylations (G-HyK and GG-HyK) in maintaining basement membrane assembly for multi-cellular life forms. The unique structural features of LHs enable remarkable stereoselectivity and regio-specificity in these critical enzymatic reactions. However, the understanding of the active-site architectures and catalytic mechanisms governing lysyl-5-hydroxylation and O-glycosylation remains limited. In this perspective, we present a comprehensive structural analysis of the intricate amino acid networks shaping the active site and secondary structure of three isoforms of LHs (LH1, LH2, and LH3), drawing comparisons with COLGALTs (COLGALT1 and COLGALT2) to enrich structural and mechanistic understandings. Overall, the detailed domain-specific structural comparisons of LH isoforms provide a strong foundation for future mechanistic studies. These insights will aid in understanding the origins of substrate specificity, elucidating regio- and stereo-selectivity, and determining the roles of specific residues in the reactivity of LH-catalyzed post-translational modifications essential for collagen biosynthesis.

Collagens constitute a superfamily of proteins that serve as the most abundant component of the extracellular matrix (ECM) (1, 2). The collagen comprises at least 28 distinct types of trimeric protein complexes encoded by 46 distinct polypeptide chains, which provide structural integrity to the ECM (Fig. 1, *A* and *B*) (3). Among the diverse types, collagen IV, a key component of the basement membrane (BM), is a primordial protein, indispensable for life forms (4), that has been biochemically characterized to undergo post-translational modifications (PTMs) in a broad range of tissues in different animals and humans (Fig. 1, *B* and *C*) (5, 6, 7). Each collagen IV chain contains a collagenous triple-helical domain more than a thousand amino acids long collagenous triple-helical domain, characterized by the canonical Gly-Xaa-Yaa repeat. This central collagenous region is flanked by the amino-terminal 7S and the carboxyl-terminal non-collagenous (NC1) domain (Fig. 1*B*). The 7S domain also undergoes O-glycosylation at hydroxylysine residues and contains a single N-glycosylated asparagine residue, which is considered essential for the proper assembly of collagen IV protomers (5, 7, 8, 9).

**Figure 1:**
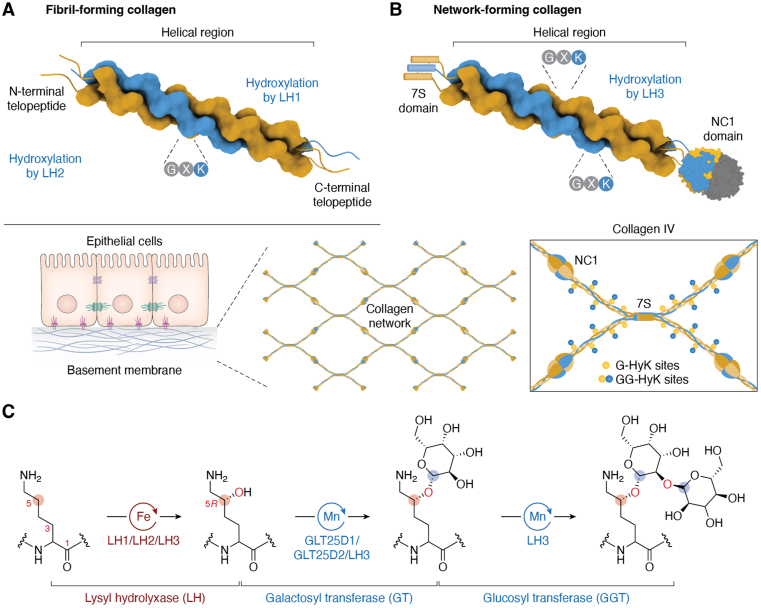
**Collagen structure and the key PTMs mediated by the complex of LH and GLT25D enzymes.***A*, a schematic structure of collagen triple helical organization, representing the triple helical and the telopeptide regions (mainly for fibrillar collagens). The activity of LH1 catalyzes the hydroxylation of Lysine residues in the triple helical region, while LH2 hydroxylates the Lysine in the telopeptide region. *B*, collagen IV, the primary abundant component of the basement membrane underlying epithelia, forms network-like structures harboring several O-glycosylated hydroxylysines in the helical region. *C*, the underlying chemical reactions of the key post-translational modifications (PTMs). The complex of LH and GLT25D enzymes performs these PTMs.

The fabrication of the basement membrane is pivotal to the evolutionary trajectory from unicellular to multicellular organisms (3, 5, 6, 10, 11, 12, 13, 14, 15, 16). The underlying pathway necessitates diverse PTMs of its constituent collagens. A particularly significant PTM involves the modification of lysine (Lys) residues located at the Yaa position of the -Gly-Xaa-Yaa-tripeptide repeat (Fig. 1*C*) (5, 17, 18). While the initial hydroxylation of these Lys residues to 5(*R*)-hydroxylysine (HyK) is a ubiquitous PTM across all collagen types, the subsequent O-glycosylation of HyK residues is distinctively regulated abundantly within collagen IV molecules of the BM (17, 18). This O-glycosylation involves the initial incorporation of galactosyl onto the HyK residues (forming galactosyl-hydroxylysine: G-HyK), followed by the addition of glucosyl molecules (forming glucosyl-galactosyl-hydroxylysine: GG-HyK) (Fig. 1*C*) (5, 19). These highly regulated PTMs are catalyzed by a complex of enzymes, including lysyl hydroxylases (LHs) and procollagen galactosyltransferases (GLT25D). While the LH enzymes catalyze the initial LH and final GGT activity, the GT activity is primarily catalyzed by the GLT25D enzyme (17, 20).

In humans, the LH enzymes are encoded by the procollagen lysyl 2-oxoglutarate-dependent dioxygenase (PLOD) gene. The LH/PLOD enzymes exist in three distinct isoforms (21): LH1/PLOD1 and LH3/PLOD3 primarily hydroxylate Lys residues in the triple-helical region, whereas LH2/PLOD2 specifically targets the telopeptide region (Fig. 1*A*) (22). Like LH/PLOD enzymes, three isoforms of GLT25D (encoded by the COLGALT gene) have been identified: GLT25D1/COLGALT1 is expressed ubiquitously, GLT25D2/COLGALT2 is localized to nervous tissue, and GLT25D3 is found in all secretory tissues. However, only GLT25D1 and GLT25D2 exhibit galactosyltransferase activity, with GLT25D3/COLGALT3 having no known role in collagen modification. Classical studies with LH3/PLOD3 knockout mice have demonstrated the importance of glycosylation of hydroxylysines in the premature aggregation of collagen IV, thereby disrupting the normal basement membrane (23, 24, 25). The glucosylation activity of LH3/PLOD3 is essential in collagen IV, as reduced GGT activity of LH3 leads to fragmentation of the basement membrane assembly, resulting in early embryonic lethality (23, 24, 25). A comparative study with LH3/PLOD3 knock-out mice and LH/PLOD mutant mice suggested that LH3 can also glycosylate lysines hydroxylated by LH1/PLOD1 and LH2/PLOD2 (23, 24, 25). In collagens, two different glycan modifications have been observed: O-glycans on hydroxylated lysine (HyK) residues and the highly conserved N-glycan in the C-terminal pro-peptide domain (8, 17, 19). Figure 2 provides a comprehensive overview of all the characterized O-glycosylation content (out of 1000 amino acids) in Collagen IV across different species (26, 27, 28, 29, 30, 31, 32, 33). Here, data on quantitative hydroxylysines and O-glycosylated lysines have been compiled in this figure from classical amino acid analyses. This provides the importance of the quantitative presence of these PTMs across different tissues and species in the context of this manuscript. However, site-specific collagen PTM characterization has remained challenging (7). Recently, a few other groups and we have established the application of high-resolution mass-spectrometry to identify and quantify site-specific collagen PTMs. Recently, we also showed that intra-tissue-specific collagen PTM heterogeneity is evident in human adrenal glands (34). ColPTMScape has also been developed as a key knowledge base to assemble and update more tissue-specific, collagen-chain-wise PTM information (35). However, mass spectrometry analysis of basement membrane collagen IV is very limited. Because collagen IV is much more glycosylated than fibrillar collagens, it poses greater analytical challenges for characterizing these modifications. Herein, we also propose that mass-spectrometry-based site-specific identification and quantitation of hydroxylysines and O-glycosylated hydroxylysine residues in collagen IV will open up new avenues in determining their role in ECM stability and cell-ECM interactions in physiological and disease conditions.

**Figure 2:**
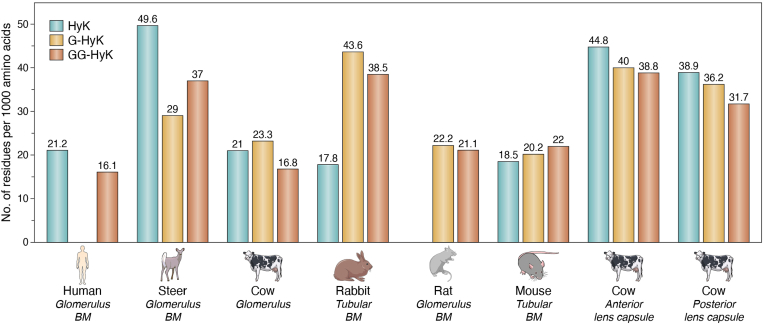
**Extent of hydroxylation and O-glycosyation across species.** The level of lysine hydroxylation and collagen O-glycosylation (out of 1000 amino acids) across species in basement membranes.

These evolutionarily conserved collagen PTM sites reflect the critical importance of physiological homeostasis. HyK residues within collagen serve as critical substrates for various PTMs, including O-glycosylation and subsequent enzymatic cross-linking mediated by lysyl oxidase (LOX). These modifications are integral to collagen biosynthesis, influencing both secretion and fibril assembly (17, 18). Additionally, detailed amino acid analysis has shown that fibrillar collagens have a lower degree of glycosylation than basement membrane (BM) collagen IV (17, 30, 36, 37). This suggests a specific distribution of the enzymes involved. Specifically, it implies that the activity of the LH3/PLOD3 isoform may be localized to BM, while the activity of LH1/PLOD1 and LH2/PLOD2 is directed toward fibrillar collagen (22, 38, 39). This difference in glycosylation patterns highlights a functional specialization among the LH isoforms, each targeting different collagen types for modification.

Despite their fundamental role in collagen maturation, the structural and mechanistic underpinnings of LHs/PLODs and GLT25Ds/COLGALTs remain poorly understood, leaving significant gaps in our knowledge of their catalytic versatility in collagen PTMs. To deepen the mechanistic understanding and clarify the basis of substrate specificity, a comprehensive structural comparison of LH/PLOD isoforms is highly desirable. Even more intriguingly, comparing the catalytic domain of LH3/PLOD3 with the collagen galactosyltransferases, GLT25D1/COLGALT1, could offer critical insights into the catalytic multifunctionality of LH3/PLOD3. To this end, this article aims to summarize recent structural developments in this domain and use these key findings to frame multiple hypotheses that lead to open questions aimed at cracking the structure-guided mechanisms of the LHs/PLODs and GLT25Ds/COLGALTs in the context of collagen PTMs. The article has been organized as follows: (i) outlook on the LH/PLOD GLT25D/COLGALT enzymatic complex, (ii) a domain-specific atomic-level structural comparison between three isoforms of LHs and between LH3 and COLGALT, and (iii) the catalytic mechanism of lysyl hydroxylation and glycosylation reactions. The thorough structural and mechanistic analyses presented in these sections have been complemented by hypotheses and open questions concerning the role of structural nuances in guiding reactivity and selectivity.

## Enzymatic complex orchestrating collagen PTMs

**Figure 3:**
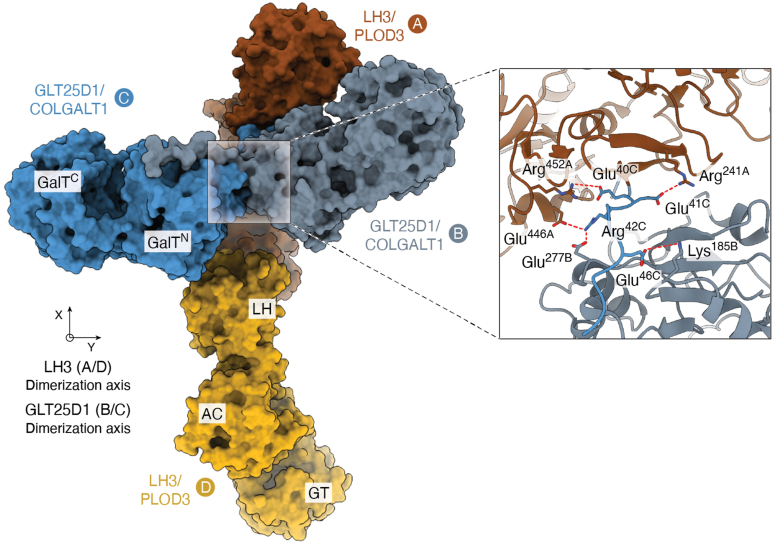
**The enymatic complex.** Tetramer of LH3/PLOD3 dimers (chains A and D, along X-axis) with GLT25D1/COLGALT1 dimers (chains B and C, along Y-axis). The residues central to tetramerization are presented in the inset. [PDB: 8ZGE].

In 2018, Federico and coworkers presented the high-resolution structural architecture of the human LH3/PLOD3 enzyme (40). Very recently, expanding on this foundational discovery, a tetrameric enzyme complex consisting of dimers of human LH3/PLOD3 and GLT25D1/COLGALT1 was simultaneously put forward by Qin, Cao, and colleagues (41), as well as Federico and coworkers (42) (Fig. 3). The discovery of the enzymatic complex serves as a foundation for the mechanistic elucidation of collagen PTMs. Its unique architecture enables the framing of a hypothesis regarding the role of this multi-enzymatic complex in substrate recognition, binding, and site-selective modifications. Recently, a thorough structural analysis of this unique enzymatic complex was presented by Kumar, Mondal, Basak, and coworkers (43). The tetrameric complex adopts a T-shaped structure with the LH3 dimers (chain A/D, Fig. 3) aligned along the X-axis and the GLT25D1 dimers (chain B/C, Fig. 3) along the Y-axis (41, 42, 43). The stability of this complex is governed by a series of electrostatic interactions at the interface between the LH3 and GLT25D1 subunits (41, 42, 43). These involve stabilizing interactions between Arg^42C^-Glu^277B^, Glu^46C^-Lys^185B^, Glu^40C^-Arg^452A^, Glu^41C^-Arg^241A^, and Arg^42C^-Glu^446A^, as highlighted in Figure 3 (41, 42, 43). LH3/PLOD3 represents the evolutionary ancestor of the LH isoforms, uniquely capable of catalyzing both GT and GGT activity alongside its primary lysyl hydroxylation function (44). The crystal structure of LH3 comprises three monodirectional domains: N-terminal galactosyl-transferase (GT) domain, central accessory (AC) domain, and the C-terminal lysyl (LH) hydroxylation (Fig. 3) (41, 42, 43). Dimerization of the LH3 occurs at the LH-domain, forming a (^N^GT-AC-^C^LH)–(^C^LH-AC-^N^GT) homodimer. In contrast to the tri-domain structure of LH3, GLT25D1/COLGALT1 has a bi-domain architecture: N-terminal GalT^N^-domain and C-terminal GalT^C^-domain, with the dimerization occurring at the GalT^N^ domain. Mutagenesis studies by Federico and coworkers have highlighted the role of GLT25D1 dimerization in forming the heteromeric complex and in collagen biosynthesis (41, 42, 43).

## Enzymatic machinery for lysine hydroxylation (LH) activity

**Figure 4:**
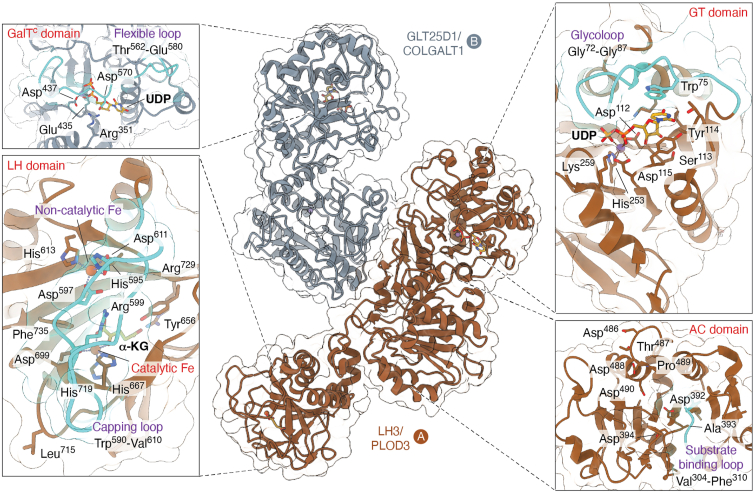
**The catalytic hubs.** Structural architecture of various domains of LH3–GLT25D1 complex: the catalytic LH-domain of LH3 (Chain A) is presented in the bottom left, and the catalytic C-domain of GLT25D1 (Chain B) is presented in the top left. The catalytic GT and non-catalytic AC-domains of LH3 are presented at the top right and bottom right, respectively.

Primary hydroxylation of collagenous lysine to 5(*R*)-hydroxylysine initiates a chain of collagen PTMs pivotal for collagen biosynthesis. This initial PTM is catalyzed by the C-terminal domain of the LH3/PLOD3 enzyme (43, 45). As an iron-dependent α-ketoglutarate (α-KG) dependent hydroxylase (46), the LH-domain contains an active site characterized by the “facial triad” (47, 48) of His^667^-His^719^-Asp^669^ residues (Fig. 4, LH-Domain). This triad coordinates the essential Fe(II) center, leaving three sites within the pseudo-octahedral coordination sphere available for exogenous ligands. The α-KG cofactor occupies two of these vacant sites, while the remaining site binds either H_2_O in the resting state or O_2_ in the active state (49, 50).

**Figure 5:**
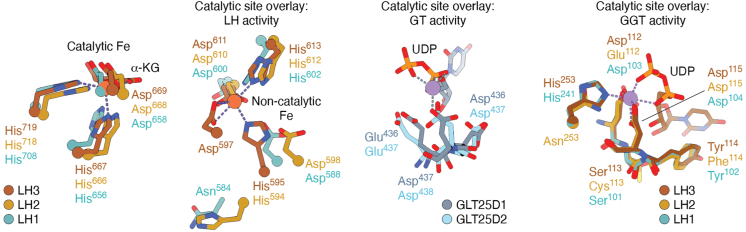
**Comparison across isoforms.** Structural overlay of the catalytic site architecture for the LH, GT, and GGT activity. [PDB: 6FXR (LH3), 8ZGE (COLGALT1), AF-O00469-F1-model_v4 (LH2), AF-Q02809-F1-model_v4 (LH1), and AF-Q8IYK4-F1-model_v4 (COLGALT2)].

The LH catalytic site is situated within a distorted barrel-like structure, the “Jelly Roll” or double-stranded β-helix (DSBH), formed by eight antiparallel β-sheets (Fig. 4, LH-Domain) (40, 51). A thorough structural comparison between the LH3 crystal structure and AlphaFold-predicted structures of LH1 and LH2 demonstrates the DSBH and the facial triad conservation across these isoenzymes The LH catalytic site is situated within a distorted barrel-like structure, the “Jelly Roll” or double-stranded β-helix (DSBH), formed by eight antiparallel β-sheets (Fig. 4, LH-Domain) (40, 51). A thorough structural comparison between the LH3 crystal structure and AlphaFold-predicted structures of LH1 and LH2 demonstrates the DSBH and the facial triad conservation across these isoenzymes (Fig. 5: LH activity) (52). Consistency of the active site residues across the LH/PLOD isoforms suggests a possible role of secondary structure of the LH domains in governing the collagen substrate specificity. Structural analysis of the LH-domain of the LH3/PLOD3 reveals a secondary, non-catalytic Fe(II) binding motif formed by His^595^-Asp^611^-Asp^597^-His^613^. When occupied by the Fe(II) ion, this locks a flexible stretch of the amino acids (Trp^590^–Val610, Fig. 4, LH Domain), the “capping loop”.

In its stable conformation, the capping loop obstructs the active site entrance by positioning the Arg^599^ residue in front of the α-KG cofactor (40, 53). Comparative analyses indicate the presence of analogous “capping loops” in both LH1 and LH2, suggesting a shared sensitivity of LH-domain catalytic activity to the binding of a second non-catalytic Fe(II) ion. Conservation of these structural features presents a profound yet underutilized opportunity for targeted drug discovery. Building on this hypothesis, recent investigations by Scietti *et al.* have focused on the design of small-molecule inhibitors targeting LH3/PLOD3 (53). Furthermore, interactions of the bound Fe(II) in the non-catalytic domain with the surrounding amino acid residues can potentially shift the Leu^715^ residue, an anchor essential for the formation of the tail-to-tail dimer of LH3 (Fig. 4, LH-Domain) (40).

## Enzymatic machinery for galactosyl-transfer (GT) activity

Besides the primary hydroxylation PTM, the resulting HyKs in collagen IV undergo further modification through a galactosyl residue, forming G-HyK. This GT-transfer activity is conventionally catalyzed by the GLT25D/COLGALT enzymes, typically belonging to the category of human galactosyltransferases. Of the GalT^N^ and GalT^C^-domains (Fig. 3), the latter is catalytically active (Fig. 4). The metal-binding site of GalT^C^ is formed by a non-canonical EDD core: Glu^435^-Asp^436^-Asp^437^(Fig. 4, GalT^C^-domain) (41, 42, 43).

Structural comparison of the GLT25D1/COLGALT1 enzyme with the AlphaFold predicted structure of GLT25D2/COLGALT2 suggests the presence of a catalytically active DxD core within the GalT^C^-domain of the second isoform (Fig. 5, GT activity) (52). The catalytically active GalT^C^-domain houses a flexible loop (Thr^562^-Glu^580^) to stabilize the UDP substrate upon binding (Fig. 4, GalT^C^-domain) (41, 42, 43). The flexible loop changes its conformation upon UDP binding, which allows the formation of a stabilizing interaction between Asp^570^ of the flexible loop and Arg^351^. This substrate-stabilizing action in the GalT^C^-domain is likely responsible for its catalytic activity (41, 42, 43). The potential Mn(II) binding motif of the GalT^N^ is also formed by the canonical DxD motif, Asp^461^-Trp^462^-Asp^463^; however, the substrate-stabilizing loop is absent in the GalT^N^ domain, rendering catalytic inactivity. While the GalT^N^ domain is catalytically dormant, through an evolutionary trajectory, this domain has been transformed into a specialized structural scaffold. Through multiple non-covalent interactions (*vide supra*), this domain provides the basis for the formation of the tetrameric LH3-GLT25D1 complex. Thus, catalytic activity has been sacrificed to achieve architectural integrity in the collagen-modifying complex.

Interestingly, the AC-domain of the LH3 holds structural similarity with the GalT^N^ domain (40, 41, 42, 43). Specifically, it features two potential Mn(II) binding motifs: a DxD motif formed by Asp^392^-Ala^393^-Asp^394^ and a DxDxD motif formed by Asp^486^-Thr^487^-Asp^488^-Pro^489^-Asp^490^ (Fig. 4, AC-domain) (40). The LH3/PLOD3 AC-domain also houses a substrate-stabilizing loop (Val^304^-Phe^310^, Fig. 4, AC-domain) next to the potential Mn(II) binding DxD motif. Despite the presence of the DxD motif and the substrate-binding loop, the AC domain is catalytically inactive (40).

## Enzymatic machinery for glucosyl-transfer (GGT) activity

Collagen IV, consisting of the G-HyK, undergoes further modification to incorporate a glucosyl residue, forming GG-HyK. The GGT transferase activity is performed by the N-terminal GT-domain of the LH3/PLOD3 enzyme (25, 40). Within LH3’s GT-domain, the active site pocket is defined by a non-canonical DxxD motif formed by Asp112 and Asp115, which, along with His^253^, collectively coordinate the Mn(II) ion (Fig. 4, GT-domain). In the LH1/PLOD1 isoenzyme, a similar facial-triad is conserved, comprising Asp^100^ and Asp^103^–part of a fully conserved version of the non-canonical DxxD motif–along with His^241^. On the other hand, the LH2/PLOD2 variant exhibits a divergence in its potential metal coordination site, which is constituted by Glu^112^, Asp^115^, and Asn^253^, thereby reflecting a notable lack of conservation concerning the anticipated DxxD motif (Fig. 5, GT-domain overlay). The lack of conservation of the Mn(II) binding motif in LH2/PLOD2, perhaps, justifies the inability of this isoform to catalyze the GGT activity.

The Mn(II) ion not only plays a vital role in catalysis but also stabilizes the pyrophosphate group present in UDP-Glucosyl substrate. Amino acid residues Gly^256^ and Lys^259^, both located within the α-helix of the C-terminal GT-domain, provide further stabilization to the pyrophosphate group by H-bonding interaction. The uracil portion of these donor substrates is stabilized by π-stacking interactions with Trp^75^ and Tyr^114^ from the DxxD motif. Trp^75^ is a part of the flexible surface loop known as the glycoloop, Gly^72^-Gly^87^ (Fig. 4, GT-domain) flexible surface loop known as the glycoloop, Gly^72^-Gly^87^. The glycoloop, like the flexible loop present in the GalT^C^-domain of the GLT25D1/COLGALT1, adopts a closed conformation upon substrate binding, further enhancing substrate stability. Mutagenesis studies targeting Trp75 and Tyr114 resulted in inactive LH3 variants, underscoring the critical role of these residues in substrate stabilization (40). Additionally, Ser^113^ and Tyr^114^, the central amino acids of the DxxD motif, stabilize the ribose hydroxyl groups by H-bonding interactions (Fig. 4, GT-domain). The conservation of the glycoloop, comprising residues Gly^61^-Gly^75^ and the DxxD motif within the LH1 isoenzyme, implies a potential stabilization of the uracil moiety and the overall substrate in this variant *via* the π-stacking interactions of Trp^64^ and Tyr^102^. The ribose hydroxyl groups may engage in hydrogen bonding interactions with Ser^101^ and Tyr^102^, central residues of the conserved DxxD motif in LH1.

## Mechanistic insights into the LH, GT, and GGT activities

The lack of structural details of human LHs has hindered progress in elucidating the intricate mechanisms underlying PTMs of lysine residues. Consequently, the molecular-level factors governing substrate specificity, regioselectivity, and stereoselectivity in the domain of human lysyl-hydroxylases have remained elusive. However, the high-resolution structural elucidation of the LH–COLGALT complex, combined with the AlphaFold structures, presents a timely opportunity to untangle these mechanistic enigmas.

**Figure 6:**
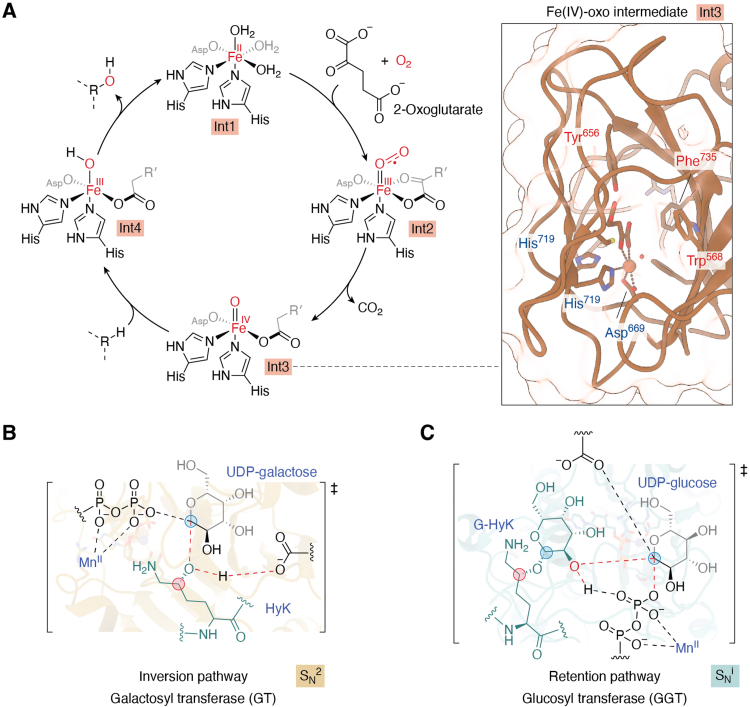
**Mechanistic details.***A*, consensus mechanism of hydroxylation involving the formation of the pivotal Fe(IV)-oxo intermediate along with the key amino acids. *B*, proposed transition states involving an SN^2^ mechanism for the GT activity, highlighting the possible role of surrounding residues. *C*, proposed transition state involving an SN^i^ pathway for the GGT activity, highlighting the possible role of surrounding residues.

As the architecture of the LH-domain of LH3 and its isoforms is similar to the family of non-heme α-KG-dependent enzymes, consisting of a facial triad of 2-His-1-Carboxylate, a consensus mechanism can be proposed for the lysyl 5-hydroxylation catalyzed by human LH isoenzymes (Fig. 6*A*) (49, 54, 55, 56). The catalytic mechanism involves the formation of a highly reactive Fe(IV)-oxo intermediate (Int3), which facilitates the hydroxylation of respective substrates through hydrogen atom transfer (HAT). The Fe(IV)-oxo intermediate featuring a high-spin (S = 2) Fe center is preceded by Fe(III)-superoxo species (Int2) formed by the initial binding of the O_2_ (57). Subsequently, a Fe(III)-hydroxo species (Int4) is formed by HAT process originating *via* the homolytic cleavage of the C^5^–H bond of lysine. Finally, the Fe center regains the initial electronic configuration of high-spin (S = 2) Fe(II) by the recombination of the R^•^ radical with the •OH radical. This consensus mechanism provides the required blueprint to address the open question of steric and electronic origins of the 5(*R*)-hydroxylation of collagenous lysine in humans. Building on recent mechanistic exploration on non-mammalian lysine hydroxylases, where an advantageously positioned tyrosine residue guided selectivity *via* a proton-coupled electron transfer (PCET) pathway. We hypothesize a similar secondary structure-guided regulation of selectivity in human LHs/PLODs. Specifically, the positioning of Tyr^656^ residue near the catalytic Fe binding site hints towards a similar “tyrosine-guided” lysine hydroxylation pathway in human collagen. A combined computational and residue-mutation-based approach will help elucidate the exact role of the LH domain's secondary structure in substrate binding and in imposing selectivity.

In contrast to the well-characterized hydroxylation mechanism, the catalytic galactosyl and glucosyl transfer processes by human LHs remain poorly understood. Although the precise role of the Mn(II) center in catalysis is unclear, it is hypothesized to contribute to substrate stabilization (58). GLT25D/COLGALT enzymes, classified as inverting glycosyltransferases, are proposed to employ an S_N_^2^-type direct displacement mechanism (Fig. 6*B*) for the formation of the glycosidic bond between 5(*R*)-hydroxylysine and UDP-galactose. A similar mechanism is also reported by Lo *et al.* in their recent exploration of human β3GalT5 (59). Conversely, the subsequent glucosylation catalyzed by LH3 is suggested to proceed *via* an S_N_^i^-type mechanism (Fig. 6*C*).

Despite these conventional mechanistic proposals, multiple open questions remain towards the catalytic GT and GGT activities. The role of the secondary structure in guiding the proposed S_N_^2^-type mechanism for GT activity, in contrast to the S_N_^i^-type mechanism for GGT activity. Furthermore, the precise role of the Mn(II) center in these catalytic mechanisms has remained elusive. Does it merely stabilize the UDP-substrate, or does it actively participate in the overall catalytic mechanism?

## Summary and outlook

In summary, this perspective article provides critical structural and mechanistic insights into the catalytic versatility of lysyl hydroxylases in collagen post-translational modifications—specifically lysyl-hydroxylation and lysyl-O-glycosylation. The intricate structural details unveiled through the LH3 crystal structure offer crucial insights into the functional capabilities and evolutionary path of the LH/PLOD isoenzyme family. Specifically, the domain-wise structural comparison between LH1, LH2, and LH3 has been presented for the first time. The variations within the GT-domain, including differences in metal coordination sites and the presence or absence of key residues, shed light on the distinct substrate preferences and catalytic mechanisms of each isoenzyme. The conservation of structural motifs, such as the DSBH and the facial triad across the LH family, underscores their crucial roles in enzymatic function. Furthermore, the identification of the unique “capping loop” mechanism and its potential regulation by a secondary Fe(II) binding site provides a deeper understanding of the modulation of LH-domain activity. Looking forward, the thorough structural and mechanistic analysis of the LH/PLOD isoforms lays the necessary foundation for several high-impact research agendas.

1. Conservation of the catalytic Fe(II) binding site across the LH/PLOD isoforms justifies their ability to modulate collagenous Lysine hydroxylation. This conservation further implicates the secondary structure of these enzymes in governing collagen substrate specificity. Further investigations must thus be led to understand the role of the molecular assembly in binding and stabilizing different collagen subtypes. This further widens the opportunity to apply high-resolution mass spectrometry in conjunction with isoform-specific knockout systems to identify the collagen-specificity of different LH isoforms.
2. The self-regulatory ability of the LH/PLOD isoforms, due to the conservation of the “capping loop” and the non-catalytic Fe(II) binding motif, offers a novel blueprint for structure-guided drug discovery to combat fibrotic diseases and cancer.
3. Future investigations should explore the "tyrosine-guided" lysine hydroxylation pathway in human collagen. Additionally, the role of “jelly roll” residues in guiding the 5(*R*) hydroxylation also needs to be unraveled.
4. The unique ability of the LH3/PLOD3 isoform to catalyze the GGT activity originates from the presence of Mn(II) binding DxxD motif along with His^253^, Trp^145^, and Trp^148^ residues. The Trp-pair crucial for UDP-substrate positioning is absent in the later LH/PLOD isoforms. The absence of these key residues may have led to the LH1/PLOD1 and LH2/PLOD2 isoforms lacking GGT catalytic activity. Further extending this structural insight, the precise catalytic role of the Mn(II) site in GGT activity remains the next enigma to be cracked.
5. The family of the GLT25D enzymes catalyzes the GT activity. The GLT25D1 and GLT25D2 are the catalytically active isoforms of this enzyme family. Though the catalytic activity is strictly restricted to the GalT^C^-domain of the dimeric enzyme, the GalT^N^-domain provides a structural basis for the formation of an enzymatic complex consisting of the LH3 and the GLTD25D1.

These findings will contribute to our growing knowledge of LH isoenzymes, paving the way for further exploration of their individual roles and therapeutic potential. The structural comparisons across the LH family provide a valuable framework for future mechanistic studies investigating their substrate specificities, catalytic activities, and potential as targets for drug development. This will further inspire the unraveling of tissue-specific collagen hydroxylysine and lysyl-O-glycosylation sites using high-resolution mass-spectrometry, which will lead to key insights into understanding the diverse nature of ECM and their role in tissue homeostasis.

## Conflict of interest

The authors declare that they have no conflicts of interest with the contents of this article.
